# Exploring the Potential Applications of Engineered Borophene in Nanobiosensing and Theranostics

**DOI:** 10.3390/bios13070740

**Published:** 2023-07-17

**Authors:** Ananya Srivastava, Daphika S. Dkhar, Nandita Singh, Uday Pratap Azad, Pranjal Chandra

**Affiliations:** 1 Department of Chemistry, Institute of Science, Banaras Hindu University, Varanasi 221005, India; ananya.gkp@gmail.com; 2 Laboratory of Bio-Physio Sensors and Nanobioengineering, School of Biochemical Engineering, Indian Institute of Technology (BHU), Varanasi 221005, India; daphikasdkhar.rs.bce21@itbhu.ac.in; 3 Department of Chemistry, Guru Ghasidas Vishwavidyalaya, Bilaspur 495009, India; singh.nanditasingh98@gmail.com

**Keywords:** borophene, nanobiosensors, biomolecules, gas, therapy

## Abstract

A monolayer of boron known as borophene has emerged as a novel and fascinating two-dimensional (2D) material with exceptional features, such as anisotropic metallic behavior and supple mechanical and optical capabilities. The engineering of smart functionalized opto-electric 2D materials is essential to obtain biosensors or biodevices of desired performance. Borophene is one of the most emerging 2D materials, and owing to its excellent electroactive surface area, high electron transport, anisotropic behavior, controllable optical and electrochemical properties, ability to be deposited on thin films, and potential to create surface functionalities, it has recently become one of the sophisticated platforms. Despite the difficulty of production, borophene may be immobilized utilizing chemistries, be functionalized on a flexible substrate, and be controlled over electro-optical properties to create a highly sensitive biosensor system that could be used for point-of-care diagnostics. Its electrochemical properties can be tailored by using appropriate nanomaterials, redox mediators, conducting polymers, etc., which will be quite useful for the detection of biomolecules at even trace levels with a high sensitivity and less detection time. This will be quite helpful in developing biosensing devices with a very high sensitivity and with less response time. So, this review will be a crucial foundation as we have discussed the basic properties, synthesis, and potential applications of borophene in nanobiosensing, as well as therapeutic applications.

## 1. Introduction

There is an ever-expanding interest in the use of 2D materials as a biosensing platform for the application of point-of-care (POC) diagnostic devices in the pursuit of patient-centric healthcare management [1,2]. Recent developments in modern technology have enabled scientists to make significant advances in the investigation of novel materials regarding their potential for use in biomedical technologies [3,4,5]. Numerous materials have been used to boost and improve the analytical parameters of biosensors used in POC. In order to identify biomolecules at ultra-low levels and in a wide range, it is necessary to design and develop high-performance analytical biosensing equipment with reduced form factors, as advised by health professionals. In clinical settings, diagnostics focused on traditional immunological instruments, such as enzyme-linked immunoassay (ELISA) and radioimmune assay, allow for the selective analysis and detection of levels of biomarkers (for example, viral immunoglobulins) in the sera of patients [6,7]. Sincere efforts have lately been made towards various synthesis methods, manufacturing, and the functionalization of 2D materials to design and build nanobiosensors and nanodevices for a variety of applications [8,9,10,11]. 2D nanomaterials such as graphene, phosphorene, MXenes, molybdenum disulphide, black phosphorus, etc., have better electrical conductivity and a higher electroactive surface area, which are the characteristics needed to immobilize biomolecules for sensing and biomedical applications. 

Recently, borophene has been classified as a sub-class of 2D materials known as Xenes, and is made up of just one element from group III to group IV that includes stanene, silicene, phosphorene, antimonene, germanene, and bismuthene [9]. Boron, an element that is immediately to the left of carbon in the periodic table, has several properties that are similar to carbon, including planar clusters [12,13,14,15,16], cage-like fullerenes [17,18,19], and one-dimensional (1D) nanotubes [20,21,22]. In the literature, boron was first reported in Sands’s work, which was published in 1957, and pure boron was first observed in 2015 [23], when silver (Ag) substrates were fruitfully used to create 2D boron sheets. The study of borophene is being pursued by the researcher in a variety of domains [24,25,26] such as nanotechnology, condensed matter, physics, chemistry, and material science. Being one atom thick, borophene is the 2D material with the lowest weight reported so far. Boron is the neighbor of carbon, so borophene has properties like graphene [27]. This resemblance encourages the researcher to use borophene for various applications. This fascinating 2D material has received more attention recently. Beyond its analogue graphene, the single sheet of boron, or borophene, is regarded as a wonder nanomaterial. In contrast to graphene, which is an isotropic material, borophene exhibits strong anisotropy because boron atoms are arranged in specific directions across its lattice structure [28]. Moreover, the direction of the edge arrangement has a significant influence on the electrical and transport characteristics of borophene. Strong anisotropic properties distinguish borophene from other materials; for instance, a borophene sheet’s Young’s modulus measured along its smoothest surface is more than that of graphene [29]. However, compared to other materials of a similar composition, borophene’s thermal properties are lower because of the dispersive effect of its phonons. This has been demonstrated by some researchers studying the thermic conductivity of borophene, who claim that it can be elucidated by its anisotropy. Scientific researchers have combined this clever and adaptable material into nanomaterials and nanotechnologies as a result of its discovery, with the hope that these products will be used to create novel biosensors, devices, and disposable medical supplies [30]. Borophene, a type of 2D material, possesses remarkable physical properties that make it highly valuable for electronic devices, biosensing, and biomedical applications [31,32]. However, it has also been recently utilized in various innovative technologies in the areas of energy conversion, nano-engineering, and photonics.

This review article presents a rigorous evaluation of 2D borophene for its use in healthcare and the biomedical sciences. Furthermore, this work covers an inclusive summary of the current relevance of borophene in various fields such as in biosensing, therapeutic applications, and bioimaging. The physical and chemical properties and the synthesis procedure are discussed. We also discuss the functionalization of borophene in developing biosensors and biosensing devices with improved analytical parameters (high sensitivity, low detection limit, low response time, etc.).

## 2. Synthesis of Borophene

**Figure 1:**
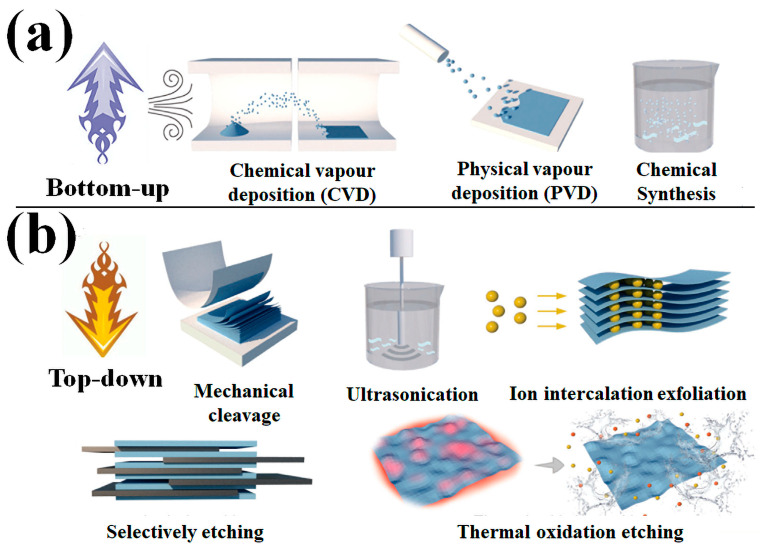
The primary approach to the fabrication of 2D nanosheets. (**a**) The bottom-up strategy via wet chemical, CVD, and PVD methods. (**b**) The top-down method includes ion intercalation exfoliation, mechanical cleavage, ultrasonication, selective etching, and thermal oxidation etching. Reproduced with permission from the authors of [12].

The two basic approaches, namely, “bottom-up” and “top-down” have been adopted for the preparation of 2D materials [33,34,35]. There are problems associated with both the bottom-up and the top-down methods [12]. Traditional bottom-up procedures like chemical vapor deposition (CVD) and physical vapor deposition (PVD) can be used to create borophene, but they are expensive, time-consuming processes that result in materials with a low surface area. Further uses in electrical devices are hindered by the difficulty of transferring borophene from metal substrates and the chances of contamination from the surrounding environment [12]. Top-down preparation saves time and money, but the resulting borophene has an inconsistent thickness, and such method is challenging to create as a single atomic layer. Hence, for the application and development of borophene, a reliable technique for the synthesis of borophene of controllable quality is much needed [12]. Vapor deposition methods such as physical and chemical vapor deposition (PVD and CVD) and wet chemical synthesis processes fall under the “bottom-up” strategy (Figure 1a) [33]. However, it must be noted that the strategies used to synthesize borophene via wet chemical methods are either in the planning stages or are only projections at this time [31]. CVD entails the reaction of associated gas phase precursors on a substrate to generate a film. Three important variables that have a significant impact on the characteristics and preparation of the products in this approach are the environment, substrates, and precursors.

In 2017, Mannix et al. [33] prepared an ultrathin monoatomic layer of borophene on silver substrate by using a high purity boron vapor of 99.9999%. Further, the synthesized material was characterized by scanning tunnelling microscopy (STM) and aberration-corrected microscopic techniques such as Auger electron spectroscopy (AES) and scanning transmission electron microscopy (STEM), which confirmed the metallic characteristics of borophene in the form of a stripe and a bandgap in the direction of the out-of-plane fold. Borophene is a 2D material that is highly anisotropic since it conducts electricity along a striped direction. Moreover, the wrinkled borophene structure possesses anisotropic mechanical properties since the Young’s modulus in the out-of-plane wrinkle direction is larger compared to graphene. Boron atoms interact strongly with one another, which is the origin of this phenomenon. Thus, it can be anticipated that 2D borophene of high mechanical strength and metallic features will play a crucial role in the applications that are associated with this topic. Mannix et al. [33] built a two-part CVD boiler for the synthesis of borophene. The copper foil used in the borophene synthesis had its grain boundaries enlarged and its surface smoothed by heating it to 1237 K before the reaction began. The ab initio density functional theory (DFT), STM, diffraction, and low-energy electron microscopy (LEEM) were used for this study. In 2019, Tai et al. [36] formulated borophene on Cu (111) and Ag (111), respectively, with an extensive investigation of its synthetic features for the two circumstances. Despite variations in the substrate temperature and other growth circumstances, it was found that the borophene domains on Ag (111) maintain their nanoscale size. Wu et al. [37] prepared borophene on an aluminium substrate in which the planar hexagonal honeycomb structure of borophene was obtained for the first time by marking a significant advance in the synthesis procedure. For the preparation of borophene, an ultrahigh vacuum environment was used to grow a monoatomic layer borophene on an Al (111) substrate using 99.9999% pure boron vapor at a deposition rate of 0.1 mL min^−1^ and at a constant temperature of 500 K. In a recent report, Kiraly et al. [38] synthesized borophene on an Au (111) substrate and investigated the effect of the substrate temperature on boron atom thermal deposition. The SEM images of the Au (111) and borophene were used to confirm the borophene synthesis on the Au, whereby the boron atoms were dissolved into gold atoms at a temperature equivalent to or exceeding 823 K, and subsequently separated onto the surface to produce borophene upon cooling. Alternatively, borophene can be synthesized on substrates such as Ag (111), Cu (111), and Al (111).

The top-down approach mainly includes mechanical cleavage [39,40,41], ultrasonication [42], ion intercalation exfoliation [43,44,45,46,47], and etching [46,47] techniques (Figure 1b). To exfoliate 2D materials from bulk materials, mechanical cleavage is commonly utilized. This approach depends on shear pressures to circumvent the van der Waals forces between the connecting layers [39,48].

**Figure 2:**
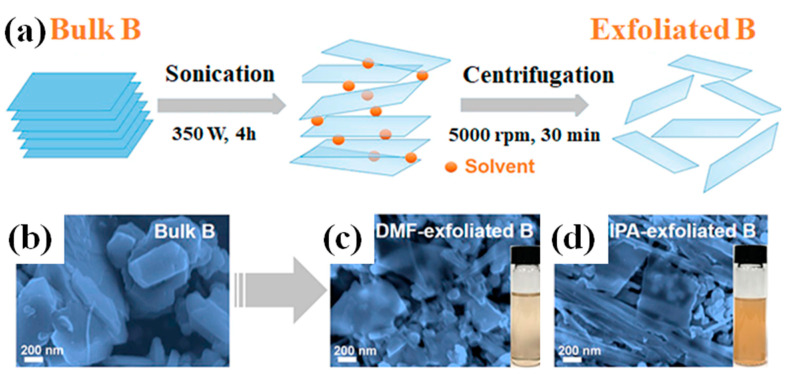
(**a**) A simplified diagram depicting the liquid-phase exfoliation process aided by sonication. It was started by sonicating bulk B powder at 350 W for 4 h in DMF/IPA (1 mg mL^−1^), where the average lateral particle size was 2 µm. Following 30 min of centrifugation at 5000 rpm, the supernatant was discarded due to the absence of unexfoliated B particles. Ultimately, this was followed by dispersions that were stable in DMF and IPA, and they were observed to have a light brown color. Exfoliation of bulk B is evident in the SEM images of (**b**) bulk B, (**c**) B sheets acquired via tip sonication in DMF, and (**d**) B sheets obtained via centrifugation at 5000 rpm for 30 min. Images of a B sheet dispersion in DMF and IPA are included as insets in figures (**c**,**d**), respectively. Reproduced with permission from the authors of [49].

By adopting the top-down approach, Li et al. [49] prepared multi-layered borophene of high quality with a tunable thickness and size using ultrasonic-assisted liquid-phase exfoliation. For the synthesis of borophene, boron powder (1 mg mL^−1^) was initially sonicated for 4 h at 350 W to create a solution in a solvent of dimethylformamide (DMF) and isopropyl alcohol (IPA), and the solution was then used in the synthesis process. By altering the speed of the centrifuge, they were able to obtain borophene of varying thicknesses (Figure 2). 

A new top-down method for synthesis involving liquid-phase stripping and high-temperature etching was developed by Ji et al. [50], who were motivated to create this method by analysing the unique chemical properties of boron, such as the resistance to oxidation while the three-dimensional bulk boron and its outer edge were both easily oxidized. Boron powder was ultrasonicated for 5 h at 500 W to be dispersed in a 1:1 solvent of N-methyl pyrrolidone (NMP) and alcohol. The boron flakes were then isolated and oxidized to B_2_O_3_ by placing them in an oxygen-containing ceramic vessel at a temperature of 923 K. Afterwards, the oxidized B_2_O_3_ was dissolved into BO_3_^3−^ via a liquid-phase exfoliation technique. By the end of the process, borophene with a thickness of 3 nm and a planar size of 110 nm was obtained.

## 3. Properties of Borophene

### Physical Properties of Borophene

When coupled to other elements, boron may develop a variety of complicated structures because it has three electrons in its outer shell. The absence of one electron in boron compared to the 2s^2^2p^2^ carbon outer shell valence suggests that a honeycomb structure like that of graphene is unstable. When boron atoms link together, the in-plane σ bonds and their antibonding are either not fulfilled or overfilled, whilst the out-of-plane π bonds and their antibonding are only partially filled. This implies that either as an acceptor or a donor, boron has a metallic property [51]. Hence, borophene has the potential to be evolved into a superb superconductor in addition to being a conductor. In a single molecule, borophene possesses a variety of band gaps of different kinds that run in distinct directions.

Functional materials are heavily influenced by their thermal characteristics. For solar or electronic equipment to remain stable and function for as long as possible, high heat conductivity is essential. Materials with a low heat conductivity, on the other hand, work well as thermoelectric and thermal insulation materials [52]. It is interesting to note that research in recent years has revealed that borophene has unique thermal transport capabilities that depend on its structure. The heat flow in a conductor with a temperature gradient is typically caused by thermal transport, but this process also contains a complicated phonon transport and scattering mechanism [53,54,55]. First-principal calculations are used to carefully examine borophene’s thermal expansion, phonon lifetimes, lattice thermal conductivity, and temperature-dependent elastic moduli. The unusually low lattice thermal conductivity of borophene is caused by strong phonon–phonon scattering. The zigzag and armchair directions of borophene exhibit impressively low thermal expansion coefficients. More startlingly, as the temperature rises, the elastic moduli are noticeably reinforced, and at about 120 K, negative in-plane Poisson’s ratios are observed along both the zigzag and armchair directions [56]. The strength of borophene was greater than that of silicene or black phosphorus when compared to the other 2D nanomaterials [57,58,59], but lesser than that of graphene [59]. This was due to the fact that the borophene B-B bonds were stronger than that of the Si-Si and P-P bonds, but weaker than the graphene C-C bonds. It is possible that this is a manifestation of metallic and covalent B-B bonds in action here. Yet, borophene might have the lowest energy level of all the 2D materials that have been researched. Moreover, due to the hexagonal structure of borophene and the hollow hexagonal appearance of borophene, the strength and strain presented by borophene are more anisotropic than those shown by graphene and silicene.

According to some studies, boron’s low atomic mass can lead to significant electron-phonon coupling, and borophene’s metallicity enables it to produce a greater carrier concentration by itself. Kou et al. found that borophene has the ability to become a superconductor due to two essential features that are commonly seen in conventional superconductors. Additionally, the surface bandgap of thicker borophene can be modified by adjusting the applied stress, as per the study conducted in [59], even though the change is minimal. As a result, the electron mobility of borophene shifts, causing a change in its conductivity type from metallic to semiconducting [12]. By simply adjusting the applied stress, the electron mobility can be increased. Thus, γ-B_28_ borophene holds potential applications in pressure-sensitive and photosensitive devices.

## 4. Chemical Properties of Borophene

2D boron, unlike its outside edge and the three-dimensional bulk of boron, has been found to be resistant to oxidation [60]. In its ground state, boron has only three valence electrons, but it can access four valence electrons by promoting one of its electrons to the 2p orbital. Since there are fewer valence electrons than valence orbitals, electron-deficient boron atoms are produced when chemical bonds are formed; thus, boron atom electrons cannot completely occupy the electronic orbitals. In order to determine the chemical constitution of the monolayer borophene, using X-ray photoelectron spectroscopy (XPS), Feng et al. [60] showed that the two low-binding energy peaks (188.2 and 187.1 eV) observed in 2D boron sheets are associated with two distinct B-B bonds, while the binding energy of the bulk boron 1s peak in the 3D bulk boron is typically within the range of 189–190 eV. As borophene has active edge states, oxidation occurs primarily at the edges, as was also discovered by Mannix et al. [24] when they exposed it to ambient conditions and looked for the oxidation state. The sections with the flawless lattice practically remain intact regardless of the large dose of oxygen [24]. Borophene is less chemically stable than other 2D materials and can easily become contaminated if exposed to air for prolonged periods. Although borophene is not as chemically stable as graphene, it helps to address the issue of 2D materials being vulnerable to oxidation.

## 5. Borophene-Based Nanosensor for Biological Molecules

**Figure 3:**
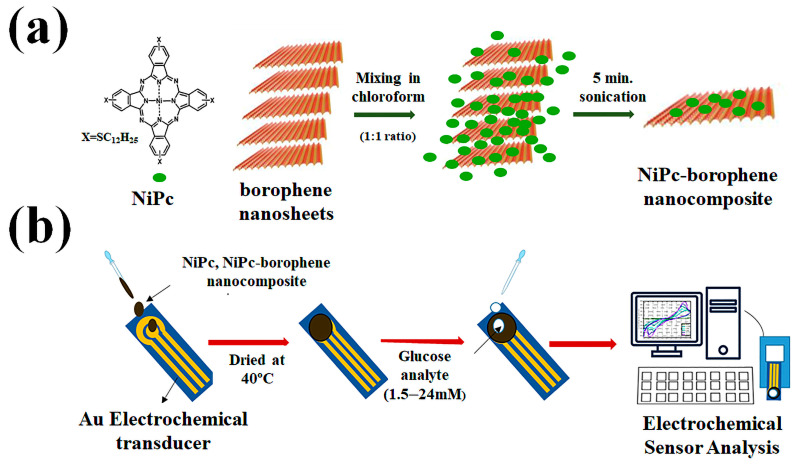
Schematics of (**a**) the fabrication of NiPc-borophene nanocomposite and (**b**) the fabrication of a non-enzymatic electrochemical sensor. Reproduced with permission from the authors of [66].

Borophene is a promising material for biosensors, owing to its exceptional physical and chemical characteristics. Considering its 2D structure and high surface-to-volume ratio, it is an excellent option for the construction of exceptionally sensitive biosensors. Borophene can also be functionalized with various biomolecules such as DNA, antibodies, and enzymes to develop specific and highly selective biosensors for detecting a wide range of analytes [61,62,63]. Additionally, borophene’s excellent electrical conductivity and optical properties make it an attractive material for developing electrochemical and optical biosensors. These unique properties make borophene a promising material for biosensing applications in healthcare, food safety, and environmental monitoring. Nevertheless, it has been demonstrated that borophene nanosheets with a high carrier mobility and outstanding chemical stability provide an excellent choice for creating nanocomposite structures with nickel phthalocyanine (NiPc) for glucose detection. The creation of temperature-independent non-enzymatic electrochemical [64] biosensors for glucose detection based on NiPc and nickel phthalocyanine-borophene nanocomposite was described by Baytemir et al. The measurements reveal that the electrical conductivities were determined to be 3 × 10^−13^ S cm^−1^ and 9.5 × 10^−9^ S cm^−1^ for the NiPc and the NiPc-borophene nanocomposites, respectively. Borophene was added to NiPc to increase its electrical conductivity (Figure 3). The sensitivity and detection limit were enhanced as a result of the borophene additive’s strong charge transport benefits. The sensitivity of the NiPc-based sensor is 0.08 µAmM^−1^ cm^−2^, whereas the sensitivity of the NiPc-borophene nanocomposite-based sensor is relatively higher at 10.31 µAmM^−1^ cm^−2^ in the linear concentration range of glucose from 1.5 to 24 mM for voltametric cycles of 60 s. The NiPc and NiPc-borophene nanocomposite-based sensors have respective detection limits of 3 µM and 0.15 µM. 

In another work, Tasaltn et al. reported a sensitive electrochemical biosensor for glucose based on polyaniline (PANI): β12 borophene that does not use enzymes [65]. Borophene-based electrochemical biosensors that do not use enzymes were looked at in detail and compared to the PANI-based biosensors that were already developed. For the PANI: β12 borophene-based biosensor, the LOD and LOQ were found to be 0.5 mM and 1.7 mM, respectively, whereas for the PANI-based electrochemical biosensor, the LOD and LOQ were determined to be 1.2 mM and 3.9 mM, respectively. The PANI: β12 borophene-based biosensor is a big step up from previous PANI-based glucose sensors. During a 1 min cyclic voltammetry test, a biosensor based on borophene showed a linear range of 1 to 12 mM glucose with a sensitivity of 96.93 µAmM^−1^ cm^−2^. The results showed that borophene-made PANI is more stable and sensitive to the presence of glucose.

Recently, Baytemir et al. [66] reported a PANI/borophene nanocomposite-based non-enzymatic electrochemical biosensor for dopamine detection. They adopted the sonication approach for the clean and affordable manufacture of borophene nanosheets. Numerous spectroscopic and microscopic techniques were used to characterize the synthesized nanocomposites. Moreover, cyclic voltammetry was utilized for the electrochemical determination of dopamine by employing the prepared PANI/borophene nanocomposite. In the presence of nanocomposite material, large peak currents were observed, showing that the addition of borophene improves the electrochemical activity of the material for sensing dopamine in the concentration range from 0.15625 to 5 µM. The PANI-based biosensor demonstrated a sensitivity of 153.67 µAµM^−1^ cm^−2^ and a LOD of 0.064 µM at a scan rate of 50 mV/s, while the PANI/borophene nanocomposite-based sensor demonstrated a sensitivity of 385.05 µAµM^−1^ cm^−2^ and a detection limit of 0.017 µM. The PANI/borophene nanocomposites showed an impressive electrochemical performance in this study, suggesting that they may be useful for dopamine investigation in the field of biomedicine.

Interestingly, a biosensor based on antimonene-borophene for sensitive detection using surface plasmon resonance (SPR) was proposed and simulated by Verma et al. [67]. The proposed sensor takes advantage of the anisotropic properties of black phosphorus/borophene and graphene/antimonene, as well as the high adsorption efficiency and delocalized orbitals of graphene/antimonene, to improve sensitivity. The optimal thickness of the gold layer was determined for the proposed biosensor, resulting in a high sensitivity of 206.26°/RIU, an extremely fine detection limit of 4.84 × 10^−6^ RIU, and a large propagation length of 3220 nm. These values are better than those of the previously reported sensors in the literature. In addition to its high sensitivity, the proposed sensor design also boasts a large penetration depth of 76.73 nm. This makes it well suited for detecting biomolecules related to DNA/RNA, as it can easily penetrate through biological membranes and reach the target molecules. Hence, it can be concluded that the antimonene-borophene-based plasmonic sensor structures have potential applications for RNA/DNA detection.

## 6. Borophene-Based Gas Nanosensor

**Figure 4:**
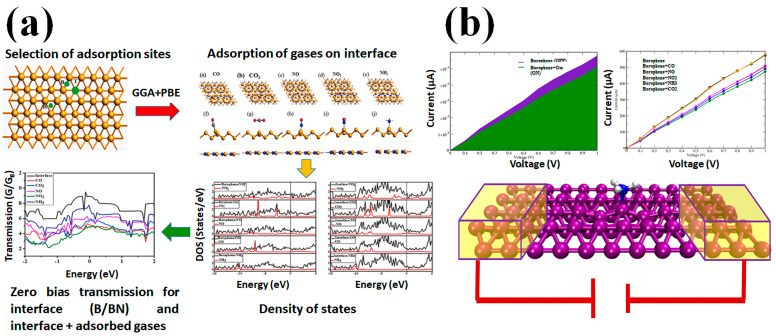
Schematic illustration of borophene-based gas sensors. (**a**) DFT analysis of the absorption sites of gases on B/BN interface followed by adsorption of the gases (a–e: top view; f–j: side view) showing density and transmission properties. Reproduced with permission from the authors of [69]. (**b**) Use of DFT and NEGF methods to determine the electrical behavior of a borophene monolayer by measuring its I-V characteristics when exposed to different gas molecules. By studying the changes in the electrical conductivity of the borophene monolayer, the presence and concentration of the adsorbed gas molecules was determined (acting as on and off states). Reproduced with permission from the authors of [70].

Gas-sensing devices are essential in various fields, but 2D materials used in previous sensors have limitations like poor air stability and a slow response time. Borophene is a new inclusion to the rapidly expanding family of 2D materials, and it shows great potential for the sensing of gases. Hou et al. demonstrated the gas-sensing potential of borophene, which was previously only predicted, by developing a chemiresistive sensor. Their study showed that the borophene-based sensor exhibited flexibility, selectivity, good sensitivity, and long-term stability, making it a promising material for gas-sensing applications. Their research filled the gap in experimental evidence for borophene’s excellent gas-sensing properties, particularly for NO_2_ [68]. The sensor has a low LOD of 200 ppb, a wide detection range from 200 ppb to 100 ppm, and rapid response/recovery times of 30 s/200 s. The sensor’s selectivity to NO_2_ was confirmed via experiments and theoretical calculations. The sensor also displayed a high stability and superior flexibility. Their findings suggest that borophene has excellent potential for high-performance sensing and detection. Similarly, in a study conducted by Khan et al. [69], it was investigated how industrial gases (CO, NO_2_, CO_2_, NH_3_, and NO) interact with the borophene-boron nitride (B/BN) interface for gas-sensing purposes using DFT and van der Waals dispersion (Figure 4a). The results show that all gases except for CO_2_ were chemisorbed on the surface, with favorable adsorption energies compared to other 2D materials. The electronic properties of the interface were improved, with a preserved metallic character after gas adsorption, as confirmed by the Hirshfeld charge analysis. The research included a thorough analysis of various factors, such as the vibrational frequencies, configurations, charge transfer, electronic density of states, adsorption energies, transmission properties, and humidity effects. This was performed in the context of designing new sensing materials for detecting environmental CH_4_, CO, and CO_2_.

In another report, Shukla et al. [70] investigated the gas-sensing properties of borophene utilizing a hybrid approach of ab initio DFT- and nonequilibrium Green’s function (NEGF)-based methods (Figure 4b). According to the study, borophene exhibits a higher affinity towards gases such as NO, CO, NH_3_, NO_2_, and CO_2_ as compared to other 2D materials like MoS_2_, phosphorene, and graphene. The calculated binding energies indicate that the affinity of borophene towards these gas molecules is significantly stronger. The stronger binding is rationalized through a charge transfer analysis. The strong binding energies of gas molecules to borophene, as compared to other 2D materials such as graphene, MoS_2_, and phosphorene, make it an excellent candidate for gas-sensing applications. The vibrational spectra of the adsorbed gases on borophene show significant shifts from their gas phase reference values, which further enhance its sensitivity for gas detection. Borophene’s metallic nature also allows for it to be used as an electrode, and the computation of the transmission function of the system (gas + borophene) shows significant changes compared to the pristine borophene surface. Moreover, the I-V characteristics of the borophene-based gas-sensing device illustrate the presence and absence of gas molecules, which can act as on and off states. Overall, the results suggest that borophene is a promising material for gas-sensing applications and is likely to attract further interest in the field. 

Using the DFT and NEGF methods, Sun et al. [71] investigated the adsorption properties of several small organic molecules on β1-borophene and their corresponding sensing applications. It was found that some molecules were physically adsorbed, while others were chemically adsorbed, causing significant changes to the chemical bonds of the adsorbed molecules. The adsorption of certain organic molecules on β1-borophene had a significant impact on its transport properties, thus making it suitable for constructing gas sensors. It was observed that β1-borophene exhibited exceptional sensitivity towards C_2_H_4_, which could be further enhanced by inducing the in-plane strain. Additionally, it was discovered that β1-borophene can form van der Waals heterostructures with select 2D semiconductors. Notably, WS_2_ acted as an excellent substrate, augmenting the thermal stability of β1-borophene and improving its sensing performance. These findings pave the way for developing borophene-based organic gas sensors. In another study, Tian et al. [72] used the DFT and NEGF methods to investigate the transport features of borophene-based nano gas sensors that incorporated gold electrodes. By investigating the impact of gas molecules on borophene in the presence of an MoS_2_ substrate and gold electrodes, the study revealed that borophene-based sensors possess the ability to detect and differentiate specific gas molecules. Moreover, the nonlinearity observed in the current voltage characteristic of the system was attributed to the presence of an MoS_2_ substrate. Additionally, the study found that gold electrodes provide charges to borophene, creating a potential barrier that leads to a decrease in the current values. These findings not only offer insights into the physics underlying the transport behaviors of 2D metallic materials with metal electrodes, but also provide valuable guidelines for designing borophene-based gas sensors.

Wang et al. designed a new potential sensor material, Cr-doped graphene-like hexagonal borophene (CrB_6_), using first-principle density functional calculations [73]. The study involved calculating the band structure, adsorption energy, density of states, charge transfer, adsorption distance, and partial density of states of CH_4_, CO, and CO_2_ gas molecules adsorbed on a monolayer of CrB_6_. The results indicate that CO adsorbs via chemisorption, while CH_4_ and CO_2_ adsorb via physisorption. The variation in the adsorption characteristics of the gas molecules has a significant impact on the density of the states and band structure of CrB_6_. These observations provide insights that can aid experimentalists in developing improved sensor materials based on hexagonal borophene.

## 7. Therapeutic and Bioimaging Applications of Borophene

Overall, the promising features of borophene make it an exceptional material for a variety of therapeutic applications beyond biosensors. Borophene has gained a lot of interest from both material and medical scientists due to its potential usefulness in the biomedical field. Another promising application is its use as a drug delivery platform. Borophene’s high surface area and tunable surface chemistry make it an attractive material for attaching and delivering drugs to specific target cells or tissues. Effortlessly studying novel nanomaterials with their applications in the biomedical area has been facilitated by the development of unique methodologies in the nanotechnology field. Boron clusters can be employed in tumor therapy using B_10_ isotopes through boron neutron capture therapy (BNCT). Ionized ^10^B, which is similar in size to tumor cells, allows for the exact removal of cancer cells without harming healthy tissue.

Gao et al. [74] utilized two types of polymeric compounds, namely, a newly synthesized PEG-polyanion and a PEG-polycation, for the formation of redox nanoparticles (BNPs). The PEG-polyanion contains a ^10^B-enriched boron cluster as a side chain in one of its segments, while the PEG-polycation has a side chain that acts as a reactive oxygen species (ROS) scavenger. The BNPs demonstrated great colloidal stability, specific accumulation, selective absorption by tumor cells, lengthy retention in tumor tissue, as well as the capacity to eliminate ROS. Following thermal neutron irradiation, the BNP-modified group showed a significant decrease in tumor growth. However, 5 ppm of ^10^B was found in the tumor tissues, while at least 20 ppm is usually needed for low molecular weight (LMW) ^10^B agents. Also, after thermal neutron irradiation, the LMW ^10^B agent-treated group had more leukocytes than the BNP-treated group, which might be because the LMW ^10^B agent can remove ROS. In the group that was treated with BNPs, there was no visible spread of tumor cells to other organs 1 month after irradiation. These results suggest that BNPs could help to improve the performance of boron neutron capture therapies (BNCTs).

**Figure 5:**
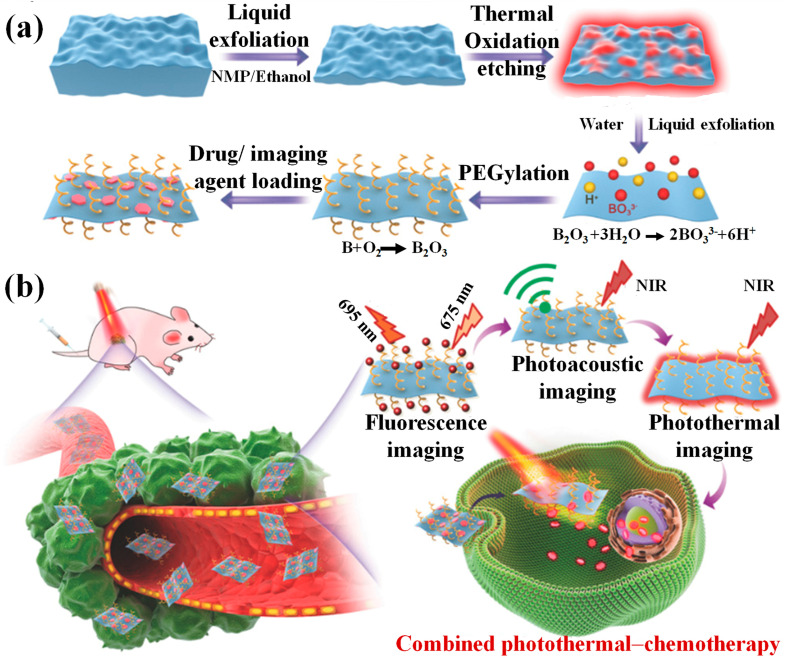
Schematic illustration of the two steps involved in creating 2D B-PEG/DOX NSs for use in multimodal imaging-guided cancer therapy: (**a**) synthesis and (**b**) systemic delivery as a photonic nanomedicine. Reproduced with permission from the authors of [50].

Ji et al. [50] proposed a new top-down strategy that combines the liquid exfoliation method with thermal oxidation etching to make high-quality ultrathin 2D boron nanosheets (2D BNSs) (Figure 5). The method used in this study involved oxidizing the B-B units on thick layers of boron at a high temperature in air, after the first liquid exfoliation, to form B_2_O_3_. During the second liquid exfoliation, B_2_O_3_ dissolved easily in water by forming BO_3_^3−^, resulting in boron nanoparticles with the desired size and thickness (100 nm for planar size and 5 nm for thickness). The boron nanoparticles were tested as new photonic drug delivery platforms for cancer imaging and therapy. To make them more biocompatible and easier to disperse, amine-functionalized polyethylene glycol (PEG-NH_2_) was electrostatically adsorbed onto the boron nanoparticles. The resulting PEGylated boron nanoparticles (B-PEG NSs) exhibited a photothermal conversion efficiency of 42.5%, making them suitable for photothermal therapy (PTT) and photothermal/photoacoustic imaging (PAI). The B-PEG NSs could also efficiently load chemotherapy drugs, for example, doxorubicin (DOX), and imaging drugs like cyanine 5.5 (Cy5.5). In vivo and in vitro experiments showed that the B-PEG NSs could be used for multimodal-imaging-guided combinatorial photothermal–chemotherapy for cancer.

The ball-milling-assisted liquid-phase exfoliation (LPE) technique was successfully used by Qi et al. [75] to manufacture borophene for use in medicine. The in vitro micro-dosimetry investigation was conducted in the presence of borophene, which allowed for the integration of spatial distribution and dose deposition. The dose enhancement ratio (DER) was quantitatively studied via a Monte Carlo simulation. Synthesized borophene was shown to be biocompatible up to a concentration of 0.2 mg mL^−1^, with less than 10% cell death at that level. The individual cellular absorption of borophene, which bypassed the nucleus but entered the cytoplasm, occurred. No appreciable shift in the DER was observed for particle therapy (PT) using proton particles. At ^10^B concentrations reaching 1 mg/g, the carbon PT DER increases by around 5%. A DER greater than 2 can be achieved via BNCT with concentrations as low as 100 µg/g. The findings of this research pave the way for the development of innovative borophene-based nanomaterials for use as radiosensitizers and imaging probes in the treatment of cancer.

**Figure 6:**
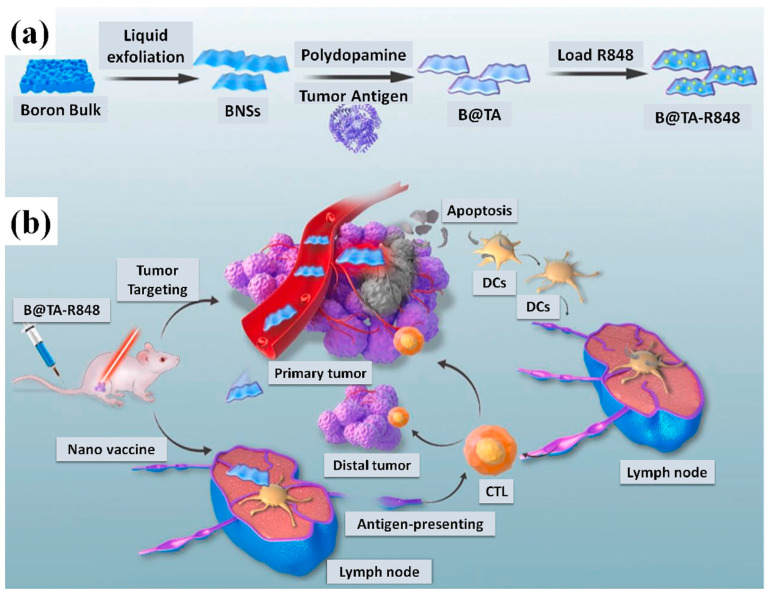
Schematic illustration showing (**a**) simplified diagram of B@TA-R848 for more precise photothermal immunotherapy. (**b**) Assembling B@TA-R848 for photo nano-vaccine B@TA-R848 administered systemically for use in multimodal imaging-guided cancer therapy (BNSs: two-dimensional boron nanosheets; TA: tumor autoantigens; DCs: dendritic cells; and CTL: cytotoxic T lymphocytes). This reprint from [76] is authorized.

The innovative personalized photonanovaccine (B@TA-R848) was built by Sun et al. [76] using TA obtained via surgery and modified on 2D boron nanosheets (BNSs) with polydopamine coating and loaded with immune adjuvant R848. B@TA-R848 possesses beneficial characteristics in terms of biocompatibility, photoacoustic imaging, photothermal action, and drug delivery and release (Figure 6). Systemic anticancer immune responses were produced, the local tumor microenvironment was altered, and the intratumoral infiltration of immune cells was increased in a mouse model of triple-negative breast cancer treated with a photonanovaccine based on B@TA-R848. To a large extent, tumor development, recurrence, and metastasis might be stifled by the combination of photo-immunotherapy. This work has significant implications for the investigation of the clinical development of tailored tumor vaccines against immunological desert cancers, since it creates a unique photonanovaccine for tumors with poor immunogenicity and high metastatic potential.

**Figure 7:**
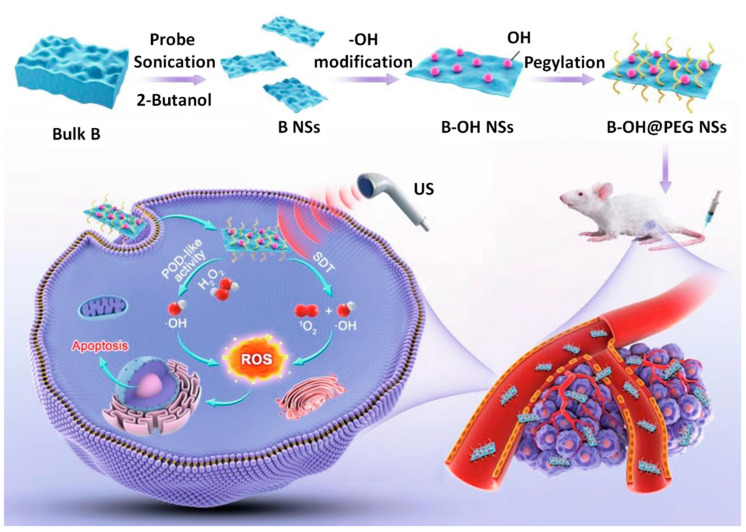
Schematic representation of the production process for BOP NSs and the mechanism underlying SDT and POD-like activity’s synergistic anti-tumor therapy. Diagram illustration is included, reproduced with permission from the authors of [77].

Recently, Xiao et al. [77] used the liquid exfoliation technique to produce boron-derived B-OH@Cy5-PEG-NH_2_ nanosheets (BOP NSs) as a novel sonosensitizer for use in sonodynamic therapy (SDT). Because of their tighter band gap in comparison to other nano-sonosensitizers, these BOP NSs reveal an increased US-stimulated severance of electron (e^−^) and hole (h^+^) pairs. In addition, the underexplored characteristics of BOP NSs, such as their appealing peroxidase-like behavior and the positive biological impacts of biodegradation, were studied. Therapeutic advantages against tumor growth were observed in in vitro and in vivo investigations, suggesting that combining ultrasound-based sonodynamic therapy (SDT) with enzyme catalytic reaction is effective in generating higher levels of reactive oxygen species (ROS). These BOP NSs are thought to be highly therapeutic in cancer therapy due to their multifunctionality and strong biodegradability, opening a new frontier for the application of 2D boron NSs (Figure 7).

**Figure 8:**
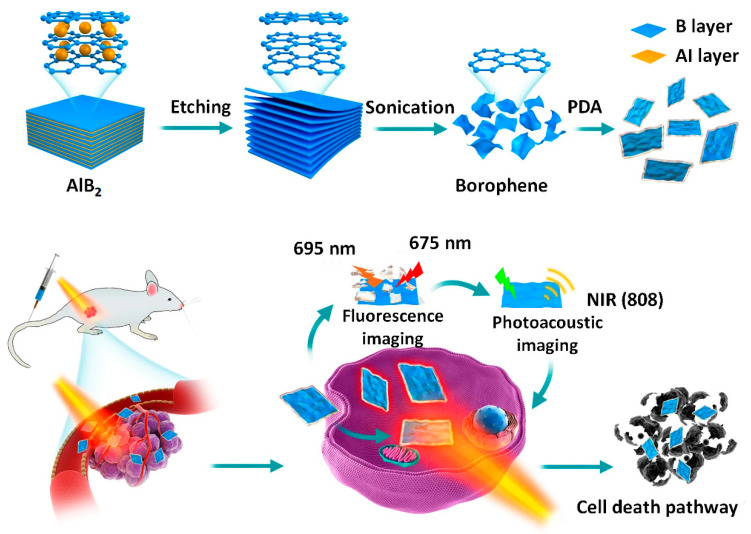
Schematic illustration of the synthesis of borophene which was prepared by etching its precursor, in the same way in which MXene was made. The surface-modified borophene was then used in multi-imaging directed photothermal therapy. Reproduced with permission from the authors of [78].

By using a straightforward selective chemical etching technique, Xie et al. [78] prepared borophene with a potent photothermal effect when exposed to near IR light. As boron lacks an electron, borophene’s photothermal efficacy is comparable to that of plasmonic Au nanoparticles, but it also has the added feature of biodegradability. Here, they showed how to convert AlB2 into ultrathin and large borophene nanosheets via selective chemical etching (thickness of 4 nm and lateral size of up to 600 nm) (Figure 8). Additionally, this novel finding reported the observation of selective acid etching behavior. In this behavior, the HCl etching of aluminum leaves behind a residual boron lattice, while HF selectively etches boron to produce an aluminum lattice.

This work illustrates that a biocompatible shrewd delivery nanoplatform of B@PDA, modified on its surface with polydopamine (PDA), can display an improved cellular absorption efficiency in response to a tumor environment. Additionally, borophene, which is biodegradable, is preferable to gold nanoparticles, which are not biodegradable, because of safety issues associated with long-term toxicity when it comes to the photothermal theranostics of large tumors employing deep penetration near IR light. This work involves the use of about 40 different types of borides; thus, it will pave the way for the further exploration of this top-down and very selective etching method to the generation of the borophene structures with rich uncharted thermal, optical, and electronic properties for numerous other technological applications.

**Figure 9:**
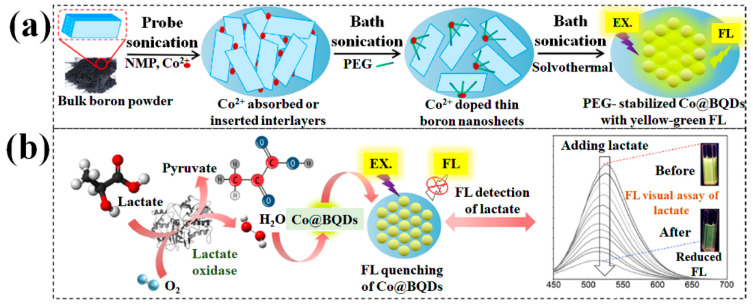
Diagrams showing (**a**) the production of Co@BQDs and (**b**) the use of an enzymatic bio-probe for FL based on Co@BQDs. Reproduced with permission from the authors of [79].

Recently, Yang et al. [79] proposed a metal-coordination technique for investigating newly discovered metal-doped boron quantum dots (Co@BQDs). It was determined that the B-Co bonding prevents the oxidation and degradation of the electronic structures of exfoliated 2D boron when exposed to oxygen. Experimental research has shown that Co^2+^ can be coordinated with exposed boron atoms by first adsorbing onto the surface of the exfoliated 2D boron. This was accomplished by adding Co^2+^ to a suspended liquid containing bulk boron, and then subjecting the liquid to probing sonication. The Co@BQDs, which are composed of Co^2+^-doped zero-dimensionalboron, were produced by subjecting exfoliated 2D boron to a solvothermal treatment. It was shown experimentally that Co@BQDs are more stable in their colloidal and FL states than BQDs used as a standard. Exfoliated boron structures are reliably stable because B-Co bonding forms to dampen the B-O reaction. For the visual FL-based imaging of solutions, as well as of solid substrates, a dispersion liquid of Co@BQDs was chosen because of how stable and brilliant their FL is. Using the FL quenching of Co@BQDs as a result of enzyme and cascade oxidation, a new FL bio-probe of lactate was investigated (Figure 9). 

This bio-probe allows for the FL-based detection of lactate in bio samples, with a wide linear detection range of 0.01-10 mM and a very low detection limit of 3.1 µM, and it displays high quantification recoveries of 98.0-102.8%. In addition, this bio-probe allowed for the flexible fluorescence-based imaging and visual measurement of lactate in both the liquid- and solid-phase systems. Such findings show that Co@BQDs have promising futures as a new class of effective imaging reagents for use in lasting tracking and bioimaging.

## 8. Conclusions and Future Prospectives

This work covers the basic information about the borophene, which is the most emerging and the lightest 2D material. In this review, along with the basic properties, the physical and chemical properties and the synthesis procedure are discussed. This review also gives the inclusive summary of the application of borophene in biosensing, biomedical applications, and bioimaging. We also discuss the functionalization of borophene to develop biosensors and biosensing devices with improved analytical parameters (high sensitivity, low detection limit, low response time, etc.). While borophene shows great potential as a material for biosensors, it is important to note that much of its potential has yet to be fully explored. Despite recent advancements in the study of borophene, its potential for biosensing applications is still relatively unexplored. One major challenge is the difficulty in synthesizing borophene with high quality and uniformity, which can impact the sensitivity and reliability of biosensors. Furthermore, while borophene has shown promise in preliminary studies, its behavior and properties in biological environments remain relatively unknown. It is still unclear how borophene will react in complex biological systems, and whether its unique properties will remain stable and effective for biosensing applications. Additionally, there is still much to be understood about the interaction between borophene and biomolecules, such as proteins and enzymes, and how this will affect the biosensor performance. Further research is necessary to fully explore the potential of borophene for biosensing applications and to develop reliable and efficient biosensors that can be used in real-world applications.
